# Academy of Stomatology

**Published:** 1898-09

**Authors:** 

**Affiliations:** Academy of Stomatology


### ACADEMY OF STOMATOLOGY.

A regular meeting of the Academy of Stomatology was held on Tuesday evening, March 22, 1898, at 1731 Chestnut Street, with the President, Professor Edwin T. Darby, in the chair.

A paper on "Restoration with Porcelain in Fracture of the Teeth" was read by Dr. Foster Jack.

(For Dr. Jack's paper, see page 493.)

At the close of the reading of Dr. Foster Jack's paper he stated that if he failed to elucidate certain parts in his discourse to the satisfaction of the members of the Society, it would give him pleasure to answer such queries as they might propound, when the following informal questioning took place:

*Dr. Huey.*—Would it not be almost impossible to preserve the pulp intact if a tooth were broken to any great extent as in such a cavity as is shown in Fig. 4?

*Dr. Foster Jack.*—No. The tooth that is broken is generally a thin tooth at the incisal edge, and the pulp does not extend far down. In a young tooth it might, but in the case cited it did not. The illustration is taken from a case in actual practice. The patient was about twelve years old.

*Dr. James Truman.*—I wish to thank Dr. Foster Jack for bringing this important subject before us. I regard it as a most important advance in dentistry and, as presented, quite original. I would inquire if Dr. Jack has tested the pins and porcelain in actual practice to know whether they are of sufficient strength? I would also like to ask how the piece is cemented to place?

*Dr. Foster Jack.*—The pins are of iridiumized platinum, and are sufficiently strong, and the pins can only be dislodged by the breaking of the tooth. As to the attachment, I cut both surfaces slightly concave. That gives a better and finer line. The porcelain corners are sufficiently strong to withstand mastication. The only danger lies in malocclusion from the lower tooth. That would sever the porcelain, but not at the point of union of the retaining porcelain with the contour portion.

*Dr. Gaskill.*—How do you fasten the two pieces of porcelain together?

*Dr. Foster Jack.*—After relating the retaining and contour porcelains with wax and trying in, they come away together. Trace around the edge of the retaining porcelain with a pencil, remove the wax and substitute a thin porcelain body, replace it in the furnace, and fuse it. The wet porcelain body holds the two portions together sufficiently to allow of a moderate amount of handling. The pieces fuse together perfectly within five minutes, and the piece is as strong at the union as at any other portion.

*Dr. Darby.*—This is a very interesting subject, and I am sure it will call forth some discussion. Dr. McQuillen has had experience with porcelain; he saw Dr. Jenkins, of Dresden, work it last summer.

DISCUSSION.

*Dr. McQuillen.*—I, unfortunately, am not skilled in the use of porcelain, but did have a great deal of pleasure from seeing Dr. Jenkins use it. He has been very successful in obtaining colors, and his method is entirely different from that shown to-night. He bakes his inlay in an impression of the cavity, and moulds up the body for a corner. He has made some wonderful and beautiful restorations, and, I believe, with almost unvarying success. His method is simply this: After preparing the cavity, he takes an impression with No. 20 or 30 gold, which he packs into the cavity with cotton, and then that is drawn out very carefully, invested in asbestos in a little platinum cup, and filled with the body. He has, I suppose, certainly fourteen or fifteen different shades that he has been able to get, and by experience he knows which one to use. After three bakings they are ready. Though these bodies are of a low fusing variety, they are not porous when fused. I remember one case of Dr. O'Brien's which included more than a third of a central incisor, and at a very slight distance it was impossible of detection.

*Dr. Darby.*—Dr. Jenkins has a little muffle made of Russia sheet-iron put on a standard and lined with asbestos. Under this little hood there is an opening, and below this a blow-pipe operated by a bellows, which throws a hot flame. Then, in a little platinum dish that has a handle, the inlay is carried into the muffle and heated up. The lining of the asbestos there retains the heat, and you get it directly on the little cap that holds the porcelain. This porcelain is in powder form and is mixed with absolute alcohol; it requires as much heat to fuse this body as it does to solder a gold plate,—almost a white heat. After baking, the body has shrunk into one corner of the impression; more is put in, and that shrinks also, so you have not your mould full. It takes about three bakings to bring it up to the desired contour, and it is important to get just body enough to be certain of a good joint at the cavity margins. Dr. Jenkins has done it so often that he almost never fails. I tried a good deal out of the mouth before I did it in. I made more failures than successes. I have put some in the mouth; probably three or four have been exceedingly satisfactory. In one or two cases I had to fit the occlusion. On masticating surfaces I have had some very excellent results.

The one thing that is lacking is a variety of shades. Dr. Jenkins expects to have about twenty shades, so that anything can be matched.

As Dr. McQuillen has stated, the operations were very beautiful, and at twelve inches from the patient's face you would hardly recognize that it was porcelain and not tooth-substance. I saw yesterday two porcelain inlays that Dr. O'Brien, Dr. Jenkins's associate, put in about two years ago, and the line of demarcation between the tooth and porcelain was so exact that even now, after two years time, one would say it was an exceedingly satisfactory operation. These fillings can be polished after grinding.

*Dr. Inglis.*—I wish to express my appreciation of the paper Dr. Jack has given us this evening. It is simply a revelation in its line to me. I have used porcelain considerably, but the ideas presented are nearly all new. I have always avoided the use of bodies which would fuse in gold, because they have been said to disintegrate in the mouth.

With regard to making the different shades, that matter is quite simple. The Wilmington Company furnished for Dr. Land a certain line of shades. One was the color of the natural gum, and five were of decided colors suited to porcelain work and continuous gum. By taking these five shades, and mixing them in various proportions, any shade can be obtained except very dark ones, and these are not ordinarily needed. These shades are yellow, white, blue, gray, and brown. I take one-half of No. 1 and one-half of No. 4; that makes a certain shade. Then I take one-fourth of No. 1 and three-fourths of No. 4; that gives me another shade; and so on. It is not necessary to keep mixtures on hand, but merely to bake a sample, which should be perforated and numbered before baking, and when it comes out from the furnace the numbers are indelibly fixed. These samples are strung on a wire. When you have a case in hand, take the shade, ring, match the tooth, and refer to the record for the proportions corresponding to the number of the shade. In order to mix bodies of different colors, one body should be freely wet with water and the other body thoroughly mixed in. The excess of water is readily absorbed with a blotter.

*Dr. McQuillen.*—I would like to say that Dr. Jenkins showed me two cases one day, one that had been in service five years in the upper cuspid on the disto-lingual surface, and there had been no disintegration. He told me it was a body with which he was not satisfied, because he found it slightly porous, and that what he was then trying to do was to make a body that would not be porous. There was another in the mouth of an Austrian general that had been in four years, and it seems to me that if porcelain will not show any disintegration in four years, it is a pretty safe thing to use.

*Dr. Inglis.*—Dr. Moffet told me that he was unable to get low fusing porcelain that would not disintegrate in the mouth, his experience having been obtained in continuous gum pieces. So I avoided its use, as I had the Land body, which was perfectly satisfactory, can be manipulated as rapidly, and is far more natural in appearance than any glass I have met with. I use that instead.

*Dr. Darby.*—It makes a great deal of difference whether this gold is backed up by asbestos investment. If you take a piece of pure gold and hold it over a flame of your blow-pipe, you will melt it; but it will stand a great deal more heat if it is covered up. The investment is in a little porcelain cap filled with pulverized asbestos and water.

*Dr. Foster Jack.*—I wish to lay stress upon the beauty of this kind of work. The pieces are cut from tooth-crowns, and it is surprising how closely one can fit a piece to a fractured tooth. None of the specimens have been touched on the outside, with the exception of the completed one. They fit nearly perfectly before you have to do any grinding on the outer surface. In the class of cases illustrated in Fig. 1, one can sometimes fit the labial surface perfectly without any reduction and without destroying the original gloss of the porcelain. I prefer the Ash crown for this work. Some of the other crowns—the old-fashioned pivot-crowns—are very good. They are sometimes better, but you cannot grind them.

*Dr. Huey.*—We have all found the Richmond solid gold crown to be unsightly in certain exposed positions. Still, it has some good characteristics. It occurred to me that a combination of the Richmond crown with porcelain would be a good one, so I devised one. Suppose that we have a bicuspid tooth with part or all of the lingual lobe remaining. This is suitably prepared to receive the crown. I then make a band of an alloy composed of twenty-two and a half parts of pure gold and one and a half parts of platinum. That is an alloy that fuses at a very much higher temperature than pure gold. I make a lap-joint and solder with pure gold. The band being accurately fitted to the tooth and allowed to extend the entire length of the intended crown, I then cut out a large part of the inciso-labial portion of the band, and between the lips formed by its mesial and distal sides I fit one of the Ash diatoric bicuspids. The tooth sets down into the band. I mix a little Timme body with water, and put a layer on the outside and inside of the band, insert the porcelain tooth, and fuse the body. If I have not here enough anchorage in the body of the tooth, I put a screw into the root, and allow it to extend up into the dovetail opening in the tooth. The cement goes all around that and holds securely. The Ash tooth is pretty strong on the grinding surface and is easily articulated, and as it is anchored on a solid bed of Harvard cement, there is nothing to give way. I have never yet had one to split. This method of crowning a bicuspid exposed to view is more satisfactory to me than any other. I can make one in an hour and a half.

*Dr. Broomell.*—It appears to me that it is not necessary to drill so far into the porcelain tips for anchorage of the pins as Dr. Jack has done. A simple covering of the head of a vulcanite pin has proved satisfactory in my hands in cases similar to these.

In a case in which the entire labial surface of an inferior incisor had been split off and the lingual portion of the crown left standing, I truncated the incisal edge and reduced the remainder to a thin wedge shape. I then made a gold cap to fit the stump of the crown, and to this fitted a cross-pin tooth in such a manner as to permit the pins to project lingually over the thin incisal edge of the cap. The tooth was then backed, related to the cap, and the piece invested and soldered.

Adjourned.

Otto E. Inglis,
*Editor Academy of Stomatology.*

